# Photodynamic Therapy in Gynecologic Malignancies: A Review of the Roswell Park Cancer Institute Experience

**DOI:** 10.3390/cancers8100088

**Published:** 2016-09-23

**Authors:** Paul C. Mayor, Shashikant Lele

**Affiliations:** Department of Surgery, Division of Gynecologic Oncology, Roswell Park Cancer Institute, Buffalo, NY 14263, USA; paul.mayor@roswellpark.org

**Keywords:** photodynamic therapy, gynecologic malignancy, vulvar, vaginal, cervical, ovarian, endometrial cancer

## Abstract

Photodynamic therapy (PDT) is a treatment modality used in the management of solid tumor malignancies that employs the use of a photosensitizing agent, a light source and oxygen in order to illicit a direct cytotoxic effect. Its use in gynecologic malignancies is somewhat novel and has been used for palliative and curative intent. At the Roswell Park Cancer Institute, the use of PDT in the management of gynecologic cancers began in the mid 1980s and since that time 35 patients have received PDT as a treatment for recurrent or metastatic cutaneous and vulvar, vaginal, anal, and cervical recurrences. In our experience, 85% patients with metastatic cutaneous lesions had a complete response. Twenty-seven percent of patients with metastatic vaginal, cervical or anal recurrences had a complete response to therapy with a median response time of 28 months. Side effects from the treatment included moderate to severe burning sensation, pain and edema at the treatment site requiring narcotic pain medication for symptom management in patients who underwent treatment to cutaneous lesions as well as lower genital tract recurrences. PDT should be considered an option in patients who are too frail to undergo the standard of care or decline the standard of care in lieu of a less invasive treatment modality.

Modern photodynamic therapy (PDT) employs the use of a photosensitizing agent, a light source and oxygen in order to illicit a treatment effect in solid tumor malignancies. The photosensitizing agent is typically injected systemically and then a delay period of 48–72 h occurs to allow the normal healthy tissue to clear the photosensitizing agent. The area with known disease burden is then exposed to a light source of specific wavelength and intensity which activates the photosensitizing agent which has accumulated within the cellular membrane causing a photodynamic reaction. This photodynamic reaction is characterized by the photochemical generation of singlet oxygen (excited state of molecular oxygen) which causes both cellular and vascular necrosis [1]. PDT not only targets tumor cells but also neo-vasculature which supplies the tumor with oxygen and nutrients. PDT induces a direct cytotoxic effect (oxidative stress) on tumor cells and initiates anti-tumor responses from the inflammatory and immune systems of the host as tumor cells are destroyed and cancer antigens are exposed [2].

The use of light as a treatment for human disease dates back to Egyptian, Indian and Chinese civilizations utilizing light to treat various diseases including rickets, vitiligo, and skin cancers [3]. However, the modern era of photodynamic therapy in the treatment of malignant neoplasms emerged in the medical literature in the 1960s after Lipson and Baldes reported that neoplastic tissue containing a photosensitizer of porphyrin mixture could fluoresce under ultraviolet light irradiation [4]. This pioneering work led to the exploration of PDT by TJ Dougherty at our institution, Roswell Park Cancer Institute (RPCI), in both clinical and pre-clinical studies with human cancers in the 1970s [5,6,7]. Dougherty’s work led to the FDA approval of the first photosensitizing agent, Photofrin, for clinical use in the 1980s [8]. Since then, RPCI and others have employed the use of photodynamic therapy as an experimental treatment for many different solid tumor malignancies. These trials have led to FDA approval for PDT in the treatment of esophageal cancer, non-small cell lung cancer and for the treatment of pre-cancerous esophageal lesions in patients with Barrett esophagus.

Historically, photodynamic therapy has been used in gynecologic malignancy with palliative and curative intent with promising results. The use of PDT as a treatment for gynecologic malignancies is a relatively novel approach with the first case series published in the literature in the mid-1980s [9]. This study was a case series of five patients which showed a mixed response to the PDT treatment. Since that time multiple case series involving patients with gynecologic malignancies have been published with reported complete response (CR) rates from 24% to 82% [1,10,11,12,13,14]. A larger case series of 21 patients by our group was published in 1989 which showed a 33% CR rate using PDT and two patients showing no evidence of disease for 28 and 36 months respectively [13]. In Germany, a case series of six patients with gynecologic malignancies showed a similar CR of 33% [12]. In 1995, a case series of 26 patients was published showing a CR of 66% (CR was designated as without symptoms) [11]. PDT has been used in the treatment of Paget’s Disease of the vulva and in one series of seven patients, the CR was 57% [14]. Our group published an update on our experience with PDT in 2013. This case series encompassed 32 patients with gynecologic malignancies and found a 82% CR rate in cutaneous metastasis and a 24% CR in patients with vaginal, cervical or anal recurrence [1].

Within the field of gynecology, PDT has not only been employed to treat malignancies but also pre-malignant lesions of the cervix. However, it is difficult to assess the efficacy of this treatment modality in this patient population as many of these lesions regress without treatment [10]. These case series have treated patients with a wide range of primary tumors including premalignant lesions of the cervix and vulva, Paget’s disease of the vulva, vulvar and vaginal carcinoma, and adenocarcinoma of the endometrium and ovary. Currently, there are no FDA approved indications for the use of PDT in gynecologic malignancies. Its use at Roswell Park Cancer Institute is limited to patients who decline traditional treatment modalities and choose to enroll in clinical studies utilizing PDT as a treatment for cutaneous and vulvo—vaginal recurrences of primary gynecologic malignancies.

As an update to the RPCI experience, we obtained Institutional Review Board approval and identified 35 patients treated with PDT by the Gynecologic Oncology service at RPCI from 1985 to 2015. These patients had primary ovarian, endometrial, vulvar, cervical, and Paget’s disease of the anal canal. These patients had multiple co-morbidities which prevented them from receiving the standard of care (surgical intervention, radiation or chemotherapy), declined the standard of care due to personal reasons, or exhausted all other treatment modalities for their cancer subtype. Patients were treated with a systemic intra-venous injection of Photofrin II at a dose of 2 mg/kg. Approximately 48 h following the injection the tumor was exposed to red light (wavelength 630 nm ± 2 nm) from a laser through a flexible four-quartz fiber with an attached lens to produce a spot of uniform intensity and/or diffuser tip fiber to uniformly illuminate the cavity (this is an existing and establish protocol that was previously developed and implemented at RPCI) [1]. This protocol allows for retreatment of lesions (with exposure to red light) within 30 days without re-administration of the Photofrin II as this drug remains within the tissue for up to 30 days. We retrospectively assessed the patient’s responses to PDT and defined complete response (CR) as a lack of detectable lesions within the area of treatment. We defined partial response (PR) as at least a 50% reduction in diameter of the lesion, and no response (NR) was defined as less than a 50% reduction in diameter of the lesion. Patients with progressive disease (PD) had enlarging of the tumor mass within the treated area. At our institution the use of PDT for gynecologic malignancies is limited to recurrences. Our experience is limited to the systemic administration of the photosensitizing agent as this is what is established in the pre-existing protocols at our institution across disease sites.

Within our cohort of 35 patients, 48 PDT treatments were administered with 28 patients receiving one treatment, five patients receiving two treatments, two patients receiving three treatments, and one patient receiving four treatments. There were nine patients with cervical cancer, eight patients with vulvar cancer (including Paget’s disease of the vulva), six with vaginal cancer, five with ovarian cancer, six with endometrial cancer, and one patient with recurrent Paget’s disease of the anal canal. Two of the patients within the cohort were excluded from evaluation. One was due to death immediately following the PDT (death attributed to Sepsis and unrelated to the PDT) and the other had insufficient clinical data. Eleven out of thirteen (85%) patients with metastatic cutaneous lesions had a CR. Six out of twenty-two (27%) patients with metastatic vaginal, cervical or anal recurrences had a CR to therapy with a median response time of 28 months. At our institution there was no significant difference in response based on primary tumor site. It was noted retrospectively that patients with lesions greater than 2 cm in depth had less of a response as the depth of penetration of the red light is about 2 cm. Fourteen patients have been treated with PDT since 2002 which was the year an electronic medical record system was implemented at our institution. Table 1 outlines the disease site, side effects and outcomes for the 14 patients that were treated at RPCI after 2002.

Side effects from the treatment included moderate to severe burning sensation, pain and edema at the treatment site requiring narcotic pain medication for symptom management in nine patients with skin lesions. These symptoms resolved within two months of treatment. Patients with vaginal recurrences had similar side effects requiring narcotic pain management for up to 3 weeks post-treatment. No fistulas occurred in this cohort secondary to tumor necrosis and no treatment related deaths occurred. No alterations in renal or hepatic function were identified.

Currently the use of PDT for gynecologic malignancies at our institute is limited to cutaneous, vaginal, vulvar, anal and cervical recurrences. The patient selected to undergo this therapy either declined the standard of care, had comorbidities that prevented them from receiving the standard of care, or exhausted all other treatment strategies for their given primary tumor. Treatment with photodynamic therapy both in the RPCI experience as well as other institutes has shown promising results in palliating symptoms or treating recurrences with complete response rates as high as 85%. The treatment strategy offers minimal side effects and can be employed in a patient population that is too frail to undergo surgery or chemotherapy. Photodynamic therapy remains an experimental treatment modality for gynecologic recurrences, however PDT should be considered an option in patients who are too frail to undergo the standard of care or decline the standard of care in lieu of a less invasive treatment modality.

## Figures and Tables

**Table 1:** Patients characteristics and outcomes from 2002 to 2016.

Cancer Category	Diagnosis	PDT Treatment Location	Side Effects	Outcomes
Cervical Cancer	Recurrent Advanced Stage Cervical Cancer	Vaginal Recurrence	Patient was lost to follow up immediately after PDT	Unknown
	Recurrent locally invasive squamous cell carcinoma of the cervix	Vaginal recurrence	None, patient tolerated PDT treatments well	PFS of 12 months and OS of 24 months
	Recurrent locally invasive squamous cell carcinoma of the cervix	Vaginal recurrence	Burning of the vulva requiring over the counter NSAIDs for treatment	PFS of 7 months and OS of 24 months
	Recurrent squamous cell carcinoma of the cervix	Vaginal recurrence	Perineal pain requiring 60 days of narcotic pain medication	PFS of 6 months and OS of 26 months
Endometrial Cancer	Recurrent FIGO Stage IB Grade II Endometrial Cancer	Vaginal Recurrence	Burning pain and swelling of the vagina and vulva requiring narcotic pain medication lasting 41 days.	PFS of 5 months OS of 23 months
	Recurrent Stage FIGO Stage IB Grade I Endometrial Cancer	Vaginal Recurrence	Unknown	PFS of 2 months and OS of over 5 years (patient currently alive)
	Recurrent FIGO Stage IA Grade 2 Endometrial Cancer	Vaginal recurrence	Patient was exposed to sunlight during the 30 days from the time of injection and experienced superficial burns to the skin requiring topical treatment	PFS and OS of at least 21 months (patient is alive today)
Ovarian Cancer	Recurrent advanced stage ovarian cancer	Vaginal recurrence	None, patient tolerated PDT treatments well	PFS and OS of over 5 years (patient is currently alive)
	Recurrent Stage FIGO Stage IIIC primary peritoneal cancer	Vaginal recurrence	Vaginal itching and discomfort not requiring medical intervention	PFS could not be determined in this patient, OS was 12 months
Vulvar Cancer	Recurrent Paget’s Disease of the Vulva	Vaginal Recurrence	None vulvar itching, pain and requiring narcotics for one month followed by Tylenol for 2 months	PFS and OS of at least 9 years, patient was lost to follow up
	Recurrent invasive squamous cell carcinoma of the vulva	Vulvar Recurrence	None, patient tolerated PDT treatments well	PFS of 2 months and OS unknown as patient lost to follow up after 5 months
	Recurrent squamous cell carcinoma of the vulvar	Vulvar recurrence	Vulvar pain and discomfort requiring oral narcotics for approximately 60 days	PFS of 12 months and OS of at least 14 months (patient is alive today)
	Recurrent Paget’s disease of the vulva	Vulvar recurrence	Vulvar swelling and pain last approximately 3 months requiring intermittent narcotic pain medication	PFS and OS of at least 15 months (patient is alive today)
Vaginal Cancer	Recurrent vaginal carcinoma in situ	Vaginal recurrence	None, patient tolerated PDT treatments well	PFS of 3 years and OS of 5 years (her death was due to medical comorbidities and was not a cancer related death)
